# Caring for frail older people in the last phase of life – the general practitioners’ view

**DOI:** 10.1186/s12904-016-0124-5

**Published:** 2016-06-02

**Authors:** Karin Geiger, Nils Schneider, Jutta Bleidorn, Katharina Klindtworth, Saskia Jünger, Gabriele Müller-Mundt

**Affiliations:** Hannover Medical School, Institute of General Practice, Hannover, Germany

**Keywords:** General practice, Primary care, Frailty, Old age, Palliative care, End-of-life care, Qualitative methods, Health services research

## Abstract

**Background:**

Frail older people are an increasingly important group in primary care due to demographic change. For these patients, a palliative care approach may be useful to sustain the quality of life in the last phase of their lives. While general practitioners (GPs) play a key role in the primary care for older patients, general palliative care is still in its infancy and little is known in Germany about caring for frail older people towards the end of life. This study aims to explore the tasks and challenges regarding the care for frail older patients in the last phase of life from the GPs’ point of view, and the latter’s perception of their own role and responsibilities.

**Methods:**

Explorative qualitative study based on semi-structured in-depth interviews with 14 GPs from urban and rural regions in Lower Saxony, Germany. Analysis was carried out according to the principles of Grounded Theory.

**Results:**

The GPs’ key commitment “caring for frail older patients until the end” as an integral part of primary care was worked out as a key category, flanked by central issues: “causal conditions and challenges,” which include patients’ preconditions and care needs as well as communication and cooperation aspects on the carers’ level. “Barriers and facilitators within the health system” refers to prerequisites of the German healthcare system, such as high caseloads. Regarding “strategies to comply with this commitment”, various self-developed strategies for the care of frail older people are presented, depending on the GPs’ understanding of their professional role and individual circumstances.

**Conclusions:**

The GPs show a strong commitment to caring for the frail older patients until the end of life. However, it is a challenging and complex task that requires significant time, which can take GPs to their limits. There is a great need to improve patient—and family-centered proactive communication, as well as interprofessional cooperation. Strengthening the team approach in primary care could relieve the burden on GPs, especially in rural areas, while simultaneously improving end-of-life care for their patients.

**Electronic supplementary material:**

The online version of this article (doi:10.1186/s12904-016-0124-5) contains supplementary material, which is available to authorized users.

## Background

Frail older people with complex care needs as a result of demographic transition and changing disease patterns are becoming an increasingly important group for primary care [1–3]. Frailty characterizes a state of increased vulnerability in older age which is often associated with multiple chronic diseases and functional limitations [4]. Older people with severe frailty are more likely to live in nursing homes, are hospitalized more often and experience a higher mortality [4–6].

The majority of frail older patients are cared for by their general practitioner (GP) in the last phase of their lives. For these patients, the development of end-of-life care plans in primary care has been advocated as an important strategy [7–9]. In a broader sense, end-of-life care has been characterized as providing palliative care in “an extended period of one to two years during which the patient/family and health professionals become aware of the life-limiting nature of their illness” [7]. Applying the key principles of palliative care, such as relieving burdensome symptoms, addressing physical, psychosocial and spiritual needs, preserving patients’ autonomy and dignity [7, 10], will support them when staying in their preferred place even when severely frail or approaching the end of their lives. In addition, the multidisciplinary team approach is viewed as essential to provide sustainable patient- and family-centered care. Such a concept has been outlined by the European Association for Palliative Care as a palliative care approach and was proposed as an appropriate concept to integrate the principles of palliative care methods and procedures in settings not specialized as palliative [7].

General practitioners and community nurses have a key role in the provision of end-of-life care in the community for patients and their families [7, 9, 11–14]. At the same time, the need for improvement of interdisciplinary collaboration, doctor–patient communication and palliative care training opportunities for primary care practitioners has been emphasized [13, 15–18]. Evidence from international research indicates that, from the GPs’ perspective, uncertainty of the illness trajectory, role ambiguity, and a lack of interprofessional collaboration and coordination in healthcare are perceived as barriers to the provision of palliative care in the community [19–21]. In addition, integrating palliative care provision into the daily routine and caseload appears to be difficult for GPs and for community nurses [21, 22].

Comprehensive end-of-life care strategies have been developed in several countries (i.e. Australia, Ireland, the Netherlands and the UK) to strengthen the delivery of primary palliative care. In the UK, for example, initiatives and tools have been developed to improve end-of-life care, among them the Gold Standards Framework to improve palliative care in general practice [23] and the Preferred Place of Care program to facilitate patients’ choice [24].

In Germany, to date, relatively little is known about the integration of palliative care into primary care [25–29]. While specialist palliative care has reached a fairly high level high level in Germany, general palliative care is still in its infancy [12, 13, 29]. Strengthening palliative care by GPs and better cooperation between primary care and specialist palliative care has been designated a public health priority in Germany [14]. However, the organizational framework and the medical compensation system in Germany are not in line with the care needs of older, chronically ill patients [30]. Public health insurance and long-term care insurance cover about 90 % of the German population. In contrast to many countries, there is no mandatory gatekeeping by GPs in the German healthcare system. This means that patients have a free choice of physicians and direct access to medical specialists of all relevant disciplines available in group or single practices as well as in hospitals. There is hardly any team approach in the community, since GPs and community nursing services are separated by different social insurance codes and financing structures. The lack of team support for GPs is aggravated by their heavy workload: GPs in Germany have a particularly high caseload (an average of approximately 250 patient contacts per physician per week) compared to international standards [31].

German GPs have been able to charge and invoice for the delivery of general palliative care via the statutory sickness insurance compensation system since 2013 [32]; however, this scheme does not define quality standards. Since palliative care has only been a compulsory subject in German medical schools since 2009, most practicing GPs have not undergone palliative training during their undergraduate medical education and it is up to them to attend palliative care courses on a voluntary basis.

In recent times, qualitative studies have focused on various aspects of primary care for older patients in Germany, such as images of old age from the GPs’ point of view [33] and the care of patients with dementia in general practice [34]. A recent study has related the GPs’ role perception to the care of older patients with complex health problems [35]. To the best of our knowledge, however, no study has yet examined the perspectives of GPs caring for frail older patients in the last phase of life within the specific context of the German healthcare system.

The project “End of Life Care for Frail Older Patients in Family Practice (ELFOP)” is a qualitative longitudinal study aimed at filling this research gap [36]. The ELFOP follows a multi-perspective approach, exploring both the perspective of patients and their relatives as well as the GP’s perspective. With respect to the GPs’ view, the study was guided by the following research questions:

- Which tasks and challenges do GPs see with regard to the care for frail older patients in the last phase of life?
- What self-awareness do GPs have regarding their own role and responsibilities in this healthcare setting?

## Methods

This paper shows GPs’ perspectives of end-of-life care for frail older patients. The data presented here originate from a broader research project comprising a qualitative prospective longitudinal study based on serial in-depth interviews with frail older patients, their informal carers and GPs over a timeframe of about 18 months. The overall background, methods and study design of the ELFOP project have been described in detail elsewhere [36].

### Recruitment

Most of the GPs who took part in the study were recruited at a local GP training course (“General Practice Day”) and via the institutes’ “Teaching Practices Network.” A purposive sampling strategy was applied to capture a multitude of different perspectives and contexts. A total of fourteen GPs were selected, following the principle of minimum–maximum contrast (including gender balance, different age groups, different degrees of practice site urbanization, and single and group practices).

### Data collection and analysis

Data collection took place by semi-structured face-to-face interviews with the GPs in their practices. Comprehensive baseline interviews were conducted consecutively between August 2012 and February 2013 (t_0_). Follow-up interviews (t_1_-t_3_) took place at six-month intervals. Thus, the data collection lasted until August 2014. The interviews were conducted by the three scientific project staff members (KG, KK, GMM). The interview guide was based on reviews of the existing literature and our own previous experience from other projects (see in detail [36])*.* The key issues of the interview guide were:

- the needs of frail older patients from the GPs’ point of view,
- the GPs’ personal experience with the care of frail older patients in their daily practice, and
- issues, challenges, and suggestions for improvement from the GPs’ point of view.

These issues were addressed both in general and specifically with regard to each of the GPs’ patients involved in the longitudinal study. For the subsequent interviews (t_1_-t_3_), the interview guide was adapted to focus on the things that had altered in the meantime.

The interviews were digitally recorded with the written informed consent of the interviewee, transcribed verbatim, completely anonymized and pseudonymized. Data analysis was performed by the three interviewers, using the MaxQDA10 software for text analysis. Analysis followed the principles of Grounded Theory by open coding and axial coding, using the code paradigm proposed by Strauss and Corbin to relate the emerging codes and (main) categories to each other [37]. Coding of the initial interviews was carried out by two members of the research team independently (KG, KK and/or GMM); codes, categories and the relationships between them was continually discussed by the team.

Quotations were translated independently by a native speaker and checked carefully by members of the research team prior to publication.

The results presented here are based on the analysis of the comprehensive initial interviews with the participating GPs (please see [38] for results concerning the patients’ views).

## Results

### Sample

Fourteen GPs with an average age of 48 years from different care regions in Lower Saxony participated in the study. The main characteristics of the GPs and their practices at baseline are shown in Table 1 (see Additional file 1: Table S1).

**Table 1:** GP Sample Characteristics (*n =* 14)

Characteristic	Details	Number (%)
Sex	male/female	7/7 (50 %)
Age	≤50 years 51 to 64 years	11 (79 %) 3 (21 %)
Experience in general practice	≤10 years 11 to 20 years	5 (36 %) 9 (64 %)
Type of Practice	Single practice Group practice	8 (57 %) 6 (43 %)
Practice location/Care region	Rural Small town Urban	5 (36 %) 4 (28 %) 5 (36 %)
Proportion of frail older patients (estimated by the GPs)	≤5 % 6 to 29 % ≥30 %	4 (28 %) 6 (43 %) 4 (28 %)

The duration of the comprehensive initial interviews varied between 20 and 113 min (with an average of 62 min). Interviews with the GPs were conducted at four time points during the study period, with two exceptions where only three interviews were realized: one GP was not able to give the first follow-up interview (t1) due to a sudden serious illness, but continued participating after recovering. Another GP took part in the study until the time of the third interview (t2), because the patients from his practice involved had died in between. Overall, 54 interviews were conducted.

### Results of the analysis

**Fig. 1:**
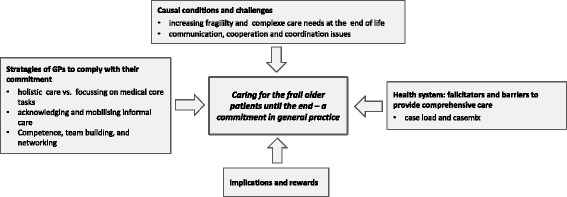
Key categories and categories

Results are presented in accordance with the coding paradigm of Strauss and Corbin [37] (Fig. 1). Starting with the key category “Caring for frail older patients until the end,” the main challenges faced by the GPs, their strategies to comply with their professional commitment, and contextual conditions, implications and rewards will be outlined.

### Caring for the frail older patients until the end—a commitment in general practice

The key phenomenon that emerged from the data is that of the GPs striving to meet their older patients’ central desire to stay in their familiar environment as long as possible, preferably until the end of life. The majority of the GPs taking part in the study regard it as their task to help realize this. Providing end-of-life care for the older patients is viewed as an integral part of primary care and a challenging but rewarding commitment in general practice:

“Actually, (I see that) also as my obligation as a GP, because they know me and so they will then also ask for me. […] So I can’t fade away when it comes to the end!” (GP 08, male, eight years of practice, group practice, rural region)

### Causal conditions and challenges

The care of frail older patients represents a particular challenge for the GPs and is influenced by a variety of considerations, particularly the complexity and fragility of the overall situation, the expectations of patients and their families, the high organizational effort, and the prevailing contextual and individual circumstances.

All in all, the interviewed GPs perceive multiple tasks regarding end-of-life care: They should try to preserve their patient’s autonomy, take into account their psychosocial and spiritual needs, treat all medical problems adequately, and accompany patients and their relatives continuously as a trusted advisor until death. The time and the effort required for these patients are generally high. Physical examinations and consultations take much longer than with younger patients, and home visits and terminal care for frail older patients are highly time-consuming, but indispensable tasks for GPs when caring for homebound patients.

#### Increasing fragility and complex needs

The care for frail older patients presents a special challenge, which is largely a result of the complexity and fragility of the overall situation. Frequently, these patients are chronically ill, if not multi-morbid, and physical functions and mobility are usually seriously compromised. The state of health of many older patients is, as a whole, fragile and can already be thrown out of balance by small “disturbances.” The care arrangements are also often fragile, for example, when the caring spouse is old and sick him/herself, as one interviewee highlighted referring to the example of an older couple in his practice:

“They [the frail couple] are at high risk of falling, highly vulnerable to everything. […] They are supported by all the props our system has. More is not possible. It’s all in a state of fragile balance!” (GP 04, male, nine years of practice, group practice, rural region)

According to the GPs’ accounts, being attentive even to minor changes in the health state and psychosocial wellbeing is essential to maintain the “fragile balance” and to prevent an institutionalization of their older patients in the last phase of their lives, or as one interviewee pointed out:

“Actually, (it’s) the attention that’s decisive, and the conversation.” (GP 11, female, sixteen years of practice, single practice, urban region)

This complex responsibility, in the interviewees’ own words described as “multilevel care,” not only implies an enormous challenge, but also lays an indispensable foundation, based on which GPs feel able to really support the patients and their family when it comes to the end.

“That means a lot of personal attention, and also to comprehensively organize everything, having everything in mind. […] Of course, in principle, they have to be accompanied to the end […] because I know them and also their needs, […] that, at the end, they’ll not have to explicitly say, but can actually, silently, presuppose that understanding.” (GP 06, female, nine years of practice, group practice, urban region)

Striving to fulfill their commitment to care for their frail older patients at home “until the end” often evokes the feeling of never having sufficient time to provide the amount of care necessary within the practice routine:

“You have the feeling, one could do more, try harder to figure out where the needs really are. […] That’s a matter of time. Because a lot of time is needed before it comes out sometimes. And to take this time, that’s often definitely the question, whether one always can.” (GP 02, male, eighteen years of practice, single practice, small town)

However, given the problem of the high workload of German GPs, the problem is to find the time to realize the palliative care approach in everyday practice, and to find the right time and sufficient calm for discussions with patients and their families about their needs and questions at the end of life, for example, patients’ preferences and possibilities for future care planning.

“It’s the ‘tyranny of the urgent’ that matters. And you are stuck with this on a daily basis. […] You have three home visits during the lunch break. […] You try somehow to handle it. You are always standing with your back against the wall.” (GP 02, male, eighteen years of practice, single practice, small town)

#### Communication, cooperation and coordination issues

A high degree of communication and coordination was reported by the interviewees to be indispensable to meet the complex needs of frail older people in general practice. Most GPs view the coordination of the complex medical care and even the initiation and coordination of care arrangements in daily living as an integral part of primary care for the older patients with complex needs. Interfaces between service providers were seen as a relevant barrier and GPs struggled with not being able to rely naturally on collaborative partnership working with hospitals, specialists or community nursing services. According to the GPs’ accounts, the cooperation within the healthcare system is highly dependent on the engagement of individual professionals or single institutions.

A key problem in the cooperation with hospitals was the discharge management, particularly when patients are discharged on a Friday afternoon, information is not adequately fed back to GPs, and care arrangements were not adapted to the patients’ actual capabilities and support needs in daily living before being discharged appropriately.

“Yes, there’s a huge dilemma […] if old people don’t have a care level assignment and are released from hospital. […] Because they don’t go into short-term care, but are discharged with an external fixator and a broken hip and sit at home and don’t know how to get [to] their bread. […] And that’s then a problem when there is no family […] around.” (GP 14, female, ten years of practice, single practice, urban region)

Regarding the collaboration with specialists, timely and sufficient communication exchange is vital, but often missing. In addition, GPs would like medical specialists to make regular home visits to immobile homebound patients so that they do not have to rely on the goodwill of individual specialists who already do so in exceptional cases.

“But if I [had], for example, a patient who has a neurological problem, a Parkinson’s patient, or even a persistent skin problem, […] it would sometimes evidently be quite good, of course, if there could be some support. If, after much effort, we manage to get the patient there [to a specialist practice], then this is obviously also very stressful for the patient.” (GP 06, female, nine years of practice, group practice, urban region)

Most of the interviewed GPs cooperate with community care services and, if necessary, inpatient care, such as short-term respite care and nursing homes. The communication varies from “not available” to a regular exchange of information, depending on the individual engagement of the GP, nurses and other professionals involved in the care for the frail older patient:

“[…] the communication depends very much on the people who work there. So, there are care services where things go quite well. […] Suddenly there’s a new nursing service, and it doesn’t work anymore.” (GP 02, male, eighteen years of practice, single practice, small town)

Regarding community nursing, varying levels of qualification and a lack of professional self-confidence are viewed as additional challenges, particularly when nurses tend to show a “safety mentality” (GP 09) and request frequent home visits in community care.

With respect to collaboration with specialist palliative care services, GPs overall reported that they felt well sustained in the care for frail older people. However, some GPs stated that the information flow in the joint care of patients could be improved, since they felt they needed to run after information which had been requested.

### Health system—and practice-related facilitators and barriers to provide comprehensive care

Whether and to what extent GPs can adequately meet the complex needs of their frail older patients is determined by specific contextual conditions within the practice and the characteristics of the care region.

#### Case load and case mix

The number of frail older patients attended by the individual GP and his/her practice team is a key factor. The amount of patients and their socio-epidemiologic profiles are influenced by various factors, such as age and social structure of the population, and the supply of physicians in the region.

Other important conditions are the *structure of the practice* (single or group practice), the infrastructure of social and health services, and the culture of cooperation of these services in the respective care region (e.g. inpatient geriatrics, medical specialists, palliative care network, nursing services, respite care, nursing homes, physiotherapists, occupational therapy, hospices).

Last but not least, *health economic factors* play an important role given the growing administrative burden and the impact of cost-saving pressures of health and social insurance. Many GPs, for example, deem physiotherapeutic interventions for frail older patients useful to prevent increasing functional impairment, but costs for long-term physiotherapy are often not covered by the health insurances without a specific medical diagnosis.

### Strategies of GPs to comply with their commitment

The GPs in this study have developed a range of different strategies for the care of frail older people depending on their own understanding of their professional role, their individual circumstances and the contextual conditions.

#### Holistic care vs. focusing on medical core tasks

The GPs’ understanding of their professional role was found to cover a wide range of views. Some of them focused on core medical tasks instead of applying a holistic approach. In this context, the role of home visits was seen differently: Whereas some GPs limit home visits only to emergency cases because of the considerable time which is needed, others are convinced of the benefits regarding care planning and inclusion of lay carers and particularly family members.

“That’s why I naturally make regular visits to these very old [patients], to see, when the time comes, which [of the family members] I may now also involve. Whom can I rely on? Firstly, to understand what the patients themselves want, but also to understand what concerns those standing beside them.” (GP 01, female, eighteen years of practice, single practice, rural region)

#### Acknowledging and mobilizing informal care

The GPs generally stress the contribution of relatives in their role as informal carers in home care. In terms of family orientation, strategies included taking into account the patient’s family status and social resources (such as informal carers), and mobilizing familial support from family or neighbors.

“We even talk with neighbors or those we know in the area […] or, in extremis, even simply with those we meet there [during the home visit at the patients’ home].” (GP 06, female, nine years of practice, group practice, urban region)

#### Competence, teambuilding and networking

Another strategy of team-oriented GPs in our sample is to improve the care of frail older patients by extending the competence of the entire practice team. The background is that (general) medical measures alone are not seen as sufficient, but rather a much broader approach is needed to meet the needs of this group of patients. One GP pointed out the benefits of practice assistants trained in wound care. Their visits replace some GP home visits and provide regular contact to the patient.

“A general practice team (with) medical assistants who would cover more of the psychosocial issues. Preventative, in the field of geriatrics, geriatric basic assessment, […], so, more foresighted, seeking to proactively help patients to respond accordingly, so that certain problems do not even occur. Medical problems, [reducing the] risk of falls of the older patients at home and everything to do with that.” (GP 03, male, eight years of practice, group practice, rural region)

Increasing delegation of certain activities and of routine home visits to appropriately trained practice assistants contributes substantially to patients’ care and relieves the GP, as has been demonstrated by the example of “specialist care assistants in general practice.”

“And we actually have very [well] qualified practice assistants who also partly take over home visits. […] And we also have three wound managers […] we are very happy, that it’s not just left to the nursing agencies, but that someone from the practice has a look at least once a week.” (GP 05, female, eleven years of practice, group practice, urban region)

Similarly, enhanced cooperation and networking with specialist palliative care services represents another strategy, which is in line with the community-based approach advocated by the World Health Organization [8]. By combining these opportunities within the German health system, some GPs succeed in building up a multi-professional network that warrants basic care for the individual frail older patient.

“One can build such a system. […] My [practice assistants] come by, I pop in, and the nurse comes. […] Thus, we almost always try to establish these three pillars for such difficult patients. […] We have regular contact with them [community nurses], so that we basically talk through each patient’s case […] look at what we decide, treatment or care pathway, […] say: watch out for this here and there, please pay attention to it. If something comes up, then report back to us. That actually works really well here.” (GP 09, male, twenty years of practice, group practice, rural region)

A subgroup among the interviewed GPs chose, as a strategy, to deliberately focus on medical aspects and clearly distance themselves from what is not considered a medical task, e.g. care coordination or the organization of technical aids.

“At least we significantly optimize the time management. Therefore, with nurses who are on hand […] and regularly look in, is there even a medical need at all? Should the pastor come, the caregiver or must a doctor?” (GP 04, male, nine years of practice, group practice, rural region)

The overall number of geriatric patients with high care needs attended by the practice can be limited, a strategy pursued by some GPs in our study, to enable them to meet the individual needs of this population despite their high overall workload. (In Germany, GPs have a certain amount of control over the number of patients for whom they are responsible. They may, for example, restrict home visits to a limited [geographical] radius or not accept responsibility for the institutional care of patients in more remote nursing homes.) This personal strategy is easier to realize in urban areas than in rural areas with a low GP density, where patients have little or no alternative in their choice of a GP.

All GPs were unanimously of the opinion that only a limited number of their patients, at any given time, can receive intensive personal care in the home environment, as is necessary in the context of primary palliative care and end-of-life care—presupposing the necessary qualifications, experience and the limited time resources available. A considerable problem is ensuring the permanent availability of care, particularly if patients are not jointly supported by specialized outpatient palliative care, since only specialized palliative care services have the resources to provide a qualified twenty-four-hour call service for seven days a week.

“Well, you obviously need to have a certain level of training in order to know about these palliative things. […] Problems are the presence, the accessibility, the visits, the effort which is also not paid for. […] But if I suddenly had five or six [dying patients], that would be too much for me. However, so far, it’s always been that there have been a maximum of two I had to look after.” (GP 08, male, eight years of practice, group practice, rural region)

Another strategy is to use particular specializations to influence the patient profile and case mix of the practice. Additional qualifications in geriatrics and palliative medicine are likely to particularly attract older and seriously ill patients, while GPs specialized in sports medicine or complementary healing methods, for example, appeal to a younger, healthier clientele.

“Through the specializations that we have, sports medicine, musculoskeletal system, preventive and alternative medicine, the target group has changed. My former colleagues who were once with me in the practice were responsible for a lot of nursing homes. Right from the outset, I did hardly any of that. […] Some [older patients] have died off and fewer took their place. As such, I guess that the percentage of the frail was significantly higher five years ago.” (GP 13, male, ten years of practice, group practice, small town)

### Implications and rewards

The challenges, frameworks and strategies for the everyday care of these patients have both positive and negative implications for GPs and practice teams. The care delivered by GPs to frail older people under current conditions has different implications for the respondents. In an ideal case, the longstanding care for frail older patients is particularly rewarding for some GPs, however, others feel more stressed or overwhelmed by this group of patients.

“It’s good to know that one [the frail older patient] can lean on this [care by their GP] to the end. And it ends with death, I know that too. But then, if I have done things properly, then I have helped to give [the patient] quality of life. That’s a good thing.” (GP 01, female, eighteen years of practice, single practice, rural region)

## Discussion

### Summary of the results

The majority of the GPs interviewed in this study try to meet the central desire of their frail older patients, and to care for them as a family until the end turned out to be a key commitment. The practical implementation is, however, experienced as a major challenge, due to preconditions and needs on the patient level, as well as communication, cooperation and coordination aspects on the carer’s level. Furthermore, barriers and facilitators are given by prerequisites of the German healthcare system. To manage these challenges, the GPs have developed their own strategies, depending on how they understand their role and individual circumstances. While some GPs find the long-term personal care of their frail older patients particularly rewarding, others feel especially burdened or overwhelmed by this patient group. Many GPs succeed in reducing both the personal workload and the psychological stress imposed by such patients by delegating, cooperating and networking, as well as by using their own additional qualifications—which simultaneously improve the quality of patient care.

The findings further suggest that proactive communication in terms of advance care planning (ACP) is not common. Even if GPs consider it useful to plan for future patient care needs, the problem remains how and when to find adequate time to discuss such issues with the patients and their families given the rush of daily practice routines.

### Comparison with the existing literature

From the perspective of the interviewed GPs, the vulnerable health status of frail older patients is characterized by the potential of being thrown off balance by even minor “disturbances.” In this context, Lee et al. described a “tipping point for a domino effect of health destabilization” [1].

Our qualitative findings confirm the results of a Belgian study, where the GPs viewed the palliative care of their patients in their home environment as a natural part of their work and experienced this aspect as quite fulfilling, but also very time-consuming and exhausting [39]. It appears partially contradictory that very few of the GPs interviewed in our study considered the need for a palliative care approach for their frail older patients. This is confirmed by Evans et al., who showed that older and infirm patients receive significantly fewer palliative medical interventions from their GPs than patients with cancer [40]. The problem areas described by Kratel regarding primary palliative care by GPs, such as the substantial time required for home visits and the fact that family doctors have to work as a “lone wolf” when palliative care support structures are missing is also reflected in our results [41].

Advance care planning was hardly ever carried out by the GPs in our study. This corresponds with the results of Glaudemans et al., who suggested that ACP has not yet found a systematic place in the outpatient care of older patients [42], and the results of Evans et al., who found that GPs are least likely to discuss ACP and questions about the end of life with older frail and demented patients compared to other groups of patients—even though people from this group are most likely to lose the capacity to make decisions [40]. The situation described by the GPs in our study regarding the difficulty of finding the right moment for such discussions in the hustle and bustle of everyday life practice—even if they are considered to be useful—is also described by De Korte-Verhoef et al., along with additional barriers affecting ACP from a GP’s point of view [43]. A German study showed the positive effects of the systematic use of ACP in the context of nursing homes [44].

Our study also indicates that the time required to coordinate the care for frail older patients and to cooperate with other professionals in the health sector is a major challenge for the surveyed GPs due to the considerable time and organizational effort, and occasionally poor communication. These results are in agreement with a study performed in the UK by Mason et al., which showed that patients with advanced, nonmalignant diseases frequently do not receive appropriate care coordination, although the needs of this patient population are particularly high [45].

Similar to the findings from a recent study by Herzog et al. [3], GPs in our study reported different views, role perceptions and action strategies regarding the primary care of older people with complex health problems. Some of the interviewees deliberately chose to limit the burden of caring for frail older people by keeping the number of patients with complex needs rather low. On the other hand, strategies to comply with the GPs’ professional commitment to care for their frail older patients until the end of life included networking and strengthening the competence of their practice staff: Some of the interviewed GPs employ practice assistants with an additional qualification to whom they delegate home visits for frail older patients, as well as other patient-related tasks, such as wound management or the implementation of generalist geriatric assessments. The GPs endorsed this model of delegating medical tasks, not only because it saves their time, but it also helps to ensure and even improve the supply of care for frail older patients in the home environment [46].

### Strengths and limitations of the study

The strength of this study is an in-depth analysis of the GP’s perspective regarding the care of frail older patients in the last phase of life using comprehensive qualitative interviews. With this approach, we have examined a hitherto neglected target group of general practice palliative care providers.

The study has several limitations. The sample size, while appropriate for qualitative enquiry, does not allow for extrapolation regarding the overall views of GPs in Germany. Even if GPs from urban and rural areas were involved, the recruitment was limited to general practices in the federal state of Lower Saxony. According to the contrast lines chosen for purposive sampling, comparative analysis of interviews showed no gender- or age-related differences. The sample probably has a positive selection bias, since it can be assumed that GPs particularly dedicated to palliative care and specifically interested in the care of older patients have participated in the study. Younger as well as older interviewees expressed a strong interest in end-of-life care issues.

## Conclusions

General practitioners show a strong commitment to caring for frail older patients until the end of life. This is a challenging and complex task that can take family doctors to the limits of their capacity, but GPs have developed certain strategies to manage these challenges individually in their practice routines. On the healthcare level, there is a significant need for improvement regarding communication and the flow of information between GPs and their various health partners, such as outpatient care, specialist doctors, hospitals and nursing homes. The delegation of activities to specifically qualified physician assistants is already partially underway, but is still underdeveloped in Germany. Strengthening the team approach in primary care could help to relieve the burden on GPs, particularly in rural areas, while simultaneously helping to improve the care for frail older patients.

## Abbreviations

ACP, advance care planning; ELFOP project, end of life care for frail older patients in family practice project; GP, general practitioner; QDA, qualitative data analysis

## Additional file

Additional file 1: Table S1 Characteristics of the participating GPs (*n =* 14) and their general practices (baseline). (DOCX 18 kb)
