# Supplementary material for: Caring for frail older people in the last phase of life – the general practitioners’ view
Source: BMC Palliat Care. 2016 Jun 2;15:52. doi: 10.1186/s12904-016-0124-5 (PMC4890268; doi:10.1186/s12904-016-0124-5)
Supplement: Additional file 1: Table S1 — Characteristics of the participating GPs (n = 14) and their general practices (baseline). (DOCX 18 kb) [file 12904_2016_124_MOESM1_ESM.docx]

**Additional file 1: Table S1:** Characteristics of the participating GPs (n=14) and their general practice (baseline)

| Code | **Sex** | **Age** | **Experience in general practice** | **Type of practice** | **Practice location / Care region** | **Proportion of frail older patients**  (estimated by the GP) |
| --- | --- | --- | --- | --- | --- | --- |
| GP 01 | female | 49 years | 18 years | single practice | rural | 11% |
| GP 02 | male | 52 years | 18 years | single practice | small town | 5% |
| GP 03 | male | 50 years | 8 years | group practice | rural | 5% |
| GP 04 | male | 47 years | 9 years | group practice | rural | 35% |
| GP 05 | female | 42 years | 11 years | group practice | urban | 12.5 |
| GP 06 | female | 40 years | 9 years | group practice | urban | 25% |
| GP 07 | female | 44 years | 11 years | single practice | urban | 5% |
| GP 08 | male | 47 years | 8 years | group practice | small town | 33% |
| GP 09 | male | 55 years | 20 years | single practice | rural | 35% |
| GP 10 | female | 42 years | 12 years | single practice | rural | 33% |
| GP 11 | female | 48 years | 16 years | single practice | urban | 8% |
| GP 12 | male | 64 years | 18 years | single practice | small town | 6% |
| GP 13 | male | 50 years | 13 years | group practice | small town | 33% |
| GP 14 | female | 43 years | 10 years | single practice | urban | 4.5% |
